# Global Research Trends in Pediatric Space Maintainers: A Bibliometric Analysis of Web of Science Literature

**DOI:** 10.7759/cureus.115531

**Published:** 2026-08-31

**Authors:** Enes Bardakci, Tuba Dogan

**Affiliations:** 1 Pediatric Dentistry, Faculty of Dentistry, Harran University, Sanliurfa, TUR; 2 Prosthodontics, Faculty of Dentistry, Harran University, Sanliurfa, TUR

**Keywords:** 3d printing, bibliometric analysis, cad/cam, children, digital dentistry, pediatric dentistry, preventive orthodontics, space maintainer

## Abstract

Background

Premature loss of primary teeth may lead to arch length reduction, space loss, and malocclusion in children, and space maintainers play an important role in preserving dental arch integrity during the mixed dentition period. Digital workflows, computer-aided design/computer-aided manufacturing (CAD/CAM), and three-dimensional (3D) printing technologies have increasingly influenced pediatric space-maintainer design and fabrication. This study aimed to map global research trends in space-maintainer research in pediatric dentistry, identify the principal contributing countries, authors, and journals, and characterize the temporal and thematic development of digital fabrication-related topics, particularly 3D printing and CAD/CAM, within this field.

Methods

A bibliometric analysis was conducted using the Web of Science Core Collection database, including articles and reviews on space maintainers and pediatric dentistry published from the database inception to 17 June 2026. Bibliometric indicators -annual production, leading countries, productive authors, core journals, keyword co-occurrence networks, and trend topics -were analyzed using Biblioshiny and VOSviewer software.

Results

A total of 221 publications specifically addressing space-maintainer appliances met the inclusion criteria. Scientific production remained limited until the mid-1990s but increased markedly after 2019, reaching its highest annual output in 2021. India was the most productive country, followed by Türkiye and Saudi Arabia. Keyword co-occurrence analysis identified five thematic clusters, with "space maintainer," "pediatric dentistry," and "premature loss" as the most frequently occurring keywords. Trend analysis showed increasing prominence of "3D printing" and "CAD/CAM"-related terms since 2021, with digital fabrication terms appearing within the core and appliance-specific thematic clusters.

Conclusions

Research on space maintainers in pediatric dentistry has expanded considerably over the last decade. The increasing visibility of 3D printing and CAD/CAM technologies indicates an emerging digital research direction toward more patient-specific space-maintainer fabrication. Future clinical trials and long-term follow-up studies are needed to evaluate the effectiveness, durability, and clinical performance of digitally manufactured space maintainers.

## Introduction

The mixed dentition period in children is a physiological process that includes the physiological resorption of primary tooth roots, the development of permanent tooth germs, jaw growth, and remodeling of the alveolar bone and soft tissues surrounding the teeth [1]. Primary teeth are responsible for maintaining the mesiodistal space required for the proper alignment of permanent teeth [2]. Early loss of primary teeth leads to migration of adjacent teeth into the extraction space, resulting in a reduction of arch length and potential impaction of permanent teeth. This reduction in arch length can lead to orthodontic crowding and malocclusion [3,4].

Early loss of primary teeth is not limited to a reduction in dental arch length. It can also lead to impaired masticatory function, phonation problems, esthetic concerns, oral hygiene difficulties, and a greater need for comprehensive orthodontic treatment later in life. For this reason, space maintainer therapy in pediatric dentistry should be regarded not merely as a mechanical method of space preservation, but as a preventive treatment approach that supports the functional, esthetic, and psychosocial well-being of children during growth and development. Appropriate space-maintainer planning during the early mixed dentition period may help permanent teeth erupt into proper position, thereby may help reduce the risk of space loss and subsequent malocclusion; however, its effect on the eventual need for comprehensive orthodontic treatment remains influenced by multiple patient and growth related fact [5,6].

Space maintainers are required to prevent space loss in the dental arch following the early loss of a primary tooth resulting from caries, trauma, ectopic eruption, congenital conditions, or arch-length abnormalities that predispose to premature root resorption [7]. Space maintainers are generally classified as fixed or removable appliances. Fixed space maintainers include various unilateral and bilateral designs, whereas removable space maintainers are predominantly acrylic-based and more dependent on patient cooperation [8].

Three-dimensional (3D) printing technology, which has a wide range of applications in orthodontics, prosthodontics, and oral and maxillofacial surgery, has also gained importance in pediatric dentistry [9]. Conventional impression-taking procedures, long clinical appointments, and repeated visits can complicate treatment in pediatric patients, particularly in younger children, those with dental anxiety, or those with limited cooperation. Digital impression systems, computer-aided design/computer-aided manufacturing (CAD/CAM) technologies, and 3D printing methods are gaining increasing attention in pediatric dentistry owing to their potential to reduce clinical chair time, improve appliance adaptation, enable digital archiving, and provide more individualized appliance fabrication [10].

Conventionally fabricated space maintainers have several reported disadvantages, including an increase in the number of oral bacteria and periodontal index scores, higher nickel sensitivity among children using nickel-containing fixed or removable appliances, fracture of fixed space maintainers, plaque accumulation, and the need for multiple appointments and laboratory stages [11]. 3D printing technologies may enable more precise and reproducible fabrication of appliances tailored to the patient's specific anatomy, and have been reported to potentially reduce clinical chair time, improve appliance adaptation, and enhance treatment comfort in pediatric patients. Certain 3D-printable materials with favorable mechanical and biocompatibility profiles have been investigated as potential alternatives for space-maintainer fabrication; however, evidence regarding their long-term clinical durability remains limited [12,13].

Bibliometric analyses allow an objective assessment of the structure, developmental direction, and future research focuses of a given field [14]. Bibliometric analyses can be conducted using complementary tools such as Bibliometrix/Biblioshiny for quantitative assessment of publication and research productivity indicators and VOSviewer for visualizing bibliometric networks, including keyword co-occurrence and thematic relationships. These tools facilitate the systematic mapping of the structure and evolution of a research field [15,16].

However, despite the increasing number of clinical and laboratory studies on conventional and digitally fabricated space maintainers, the global evolution, intellectual structure, geographical distribution, and emerging research themes in this field have not yet been systematically characterized using bibliometric methods. In particular, the extent to which digital fabrication technologies, such as 3D printing and CAD/CAM, have emerged within literature regarding space maintainers remains unclear. To the best of our knowledge, and based on our review of the available literature, this study represents one of the first comprehensive bibliometric analyses specifically focused on space maintainers in pediatric dentistry. The present study provides a comprehensive bibliometric assessment covering the indexed literature from 1982 to 2026 and combines descriptive bibliometric indicators, keyword co-occurrence analysis, trend-topic analysis, and evaluation according to Lotka’s and Bradford’s laws. Therefore, this study aimed to map the global research landscape of pediatric space maintainers using bibliometric methods and to characterize research trends related to digital fabrication technologies, particularly 3D printing and CAD/CAM, within this field.

## Materials and methods

Data source and search strategy

A comprehensive literature search was performed using the Web of Science (WoS) Core Collection to map the international research landscape concerning space maintainers in pediatric dentistry. The WoS Core Collection was selected due to its standardized indexing, consistent with established multi-database bibliometric appraisal frameworks in orthodontic research [17]. The analysis was further restricted to the Science Citation Index Expanded (SCI-Expanded) and Emerging Sources Citation Index (ESCI) collections to maintain a consistent bibliographic framework and focus on indexed peer-reviewed scholarly publications.

The use of WoS was intended to provide a reproducible and internationally standardized bibliographic dataset rather than to capture every publication worldwide. Accordingly, the term “global” in the present study refers to the international research landscape represented within the WoS Core Collection. Publications indexed exclusively in other databases, including Scopus, PubMed/MEDLINE, Embase, or regional bibliographic databases, may not have been captured. Therefore, the findings should not be interpreted as an exhaustive census of all worldwide publications on pediatric space maintainers.

No time restriction was applied in order to capture the historical development of the literature from the earliest available record. Data were downloaded from the WoS database on 17 June 2026, and publications added after this date were not included in the analysis.

The advanced search strategy was performed using the WoS Core Collection Topic field (TS=), as follows:

TS=((“space maintainer” OR “space maintenance” OR “space retention” OR “lingual arch” OR “band and loop*” OR “distal shoe*” OR “nance appliance*”) AND (“pediatric dentistry” OR “pedodontic*” OR “primary dentition” OR “primary teeth” OR “deciduous teeth” OR “mixed dentition” OR “child*” OR “premature loss”))**

The search strategy was intentionally designed to maximize sensitivity and capture the broad spectrum of publications related to space maintainers in pediatric dentistry. Consequently, some publications addressing closely related topics, such as mixed dentition, lingual arch, and space retention, were initially retrieved and subsequently evaluated during the screening process described below.

Because all records were retrieved from a single database in a single export, duplicate records were not expected; nevertheless, the exported file was checked for duplication before screening. Duplicate detection was performed using the DOI field as the primary key, supplemented by matching on the WoS accession number and on the combined title-publication year string for records without a DOI. No duplicate records were identified, and this check was completed prior to the title-and-abstract screening.

Inclusion and exclusion criteria

To preserve scientific quality and accredited peer-review standards, document types were strictly limited to original "Article" and "Review". Other document types, such as editorial letters, conference abstracts, book chapters, and letters to the editor, were excluded from the analysis. To ensure bibliographic consistency, only publications indexed in SCI-Expanded and ESCI were included. Application of these database filters yielded 293 candidate records. These records were then independently screened for topical relevance to space maintainers in pediatric dentistry by two reviewers (E.B. and T.D.); no disagreements arose between reviewers, and this screening excluded 6 records, resulting in a preliminary dataset of 287 eligible documents. Because the search strategy was intentionally broad and also captured studies on dental trauma, crossbite correction, and maxillary protraction that share terminology (e.g., “lingual arch,” “Nance appliance,” and “transpalatal arch”) but do not concern space-maintainer therapy itself, a second, appliance-specific screening stage was applied to the preliminary dataset. Unlike the first-stage screening described above, this second stage was performed by a single reviewer (E.B.) using pre-specified title/abstract criteria, rather than as an independent dual-reviewer assessment; this methodological difference is acknowledged as a limitation.

If studies discussed purposes other than space maintenance, their presence in the search results was not considered sufficient for inclusion. During the appliance-specific screening stage, records containing these terms were retained only when the title or abstract explicitly indicated a space-management context, including premature loss of primary teeth, space loss, leeway space, extraction space, or preservation of arch space. Records describing these appliances primarily as active orthodontic devices for purposes unrelated to space preservation - such as molar distalization, transverse expansion, crossbite correction, maxillary protraction/Class III treatment, or orthodontic anchorage - were excluded. Records in which a space-maintainer term occurred only incidentally within studies focused on another clinical topic were also excluded because terms such as “lingual arch,” “Nance appliance,” and “transpalatal arch” may describe the appliances used.

This second appliance-specific screening stage excluded 66 records, reducing the preliminary dataset from 287 to 221 eligible documents. Of the 66 records excluded at this stage, 40 were removed because the space-maintainer term was incidental to a study whose primary clinical focus was different, such as dental trauma, pulp therapy, restorative materials, treatment under general anesthesia, dental education, or a syndrome-related case report, or because a space maintainer was one of several applications presented rather than the primary appliance-specific focus of the study; 22 were excluded because the primary focus was active orthodontic or interceptive treatment rather than space preservation, including distalization, crossbite correction, maxillary protraction, expansion, interceptive extraction, and the use of a lingual arch, Nance appliance, or transpalatal arch as an active tooth-movement, functional, or eruption-guidance device; and four were excluded because they concerned mixed-dentition space analysis or prediction equations rather than a space-maintainer appliance or its clinical use.

Records meeting more than one criterion were assigned to the first applicable category in the order given above, so that the categories were mutually exclusive and summed to the stage total. The final dataset therefore consisted only of studies explicitly addressing space-maintainer appliances or their clinical use within a space-management context. The final dataset was downloaded in Plain Text (UTF-8) format using the “Full Record and Cited References” option. The overall study selection and screening process is summarized in Figure 1.

**Figure 1:**
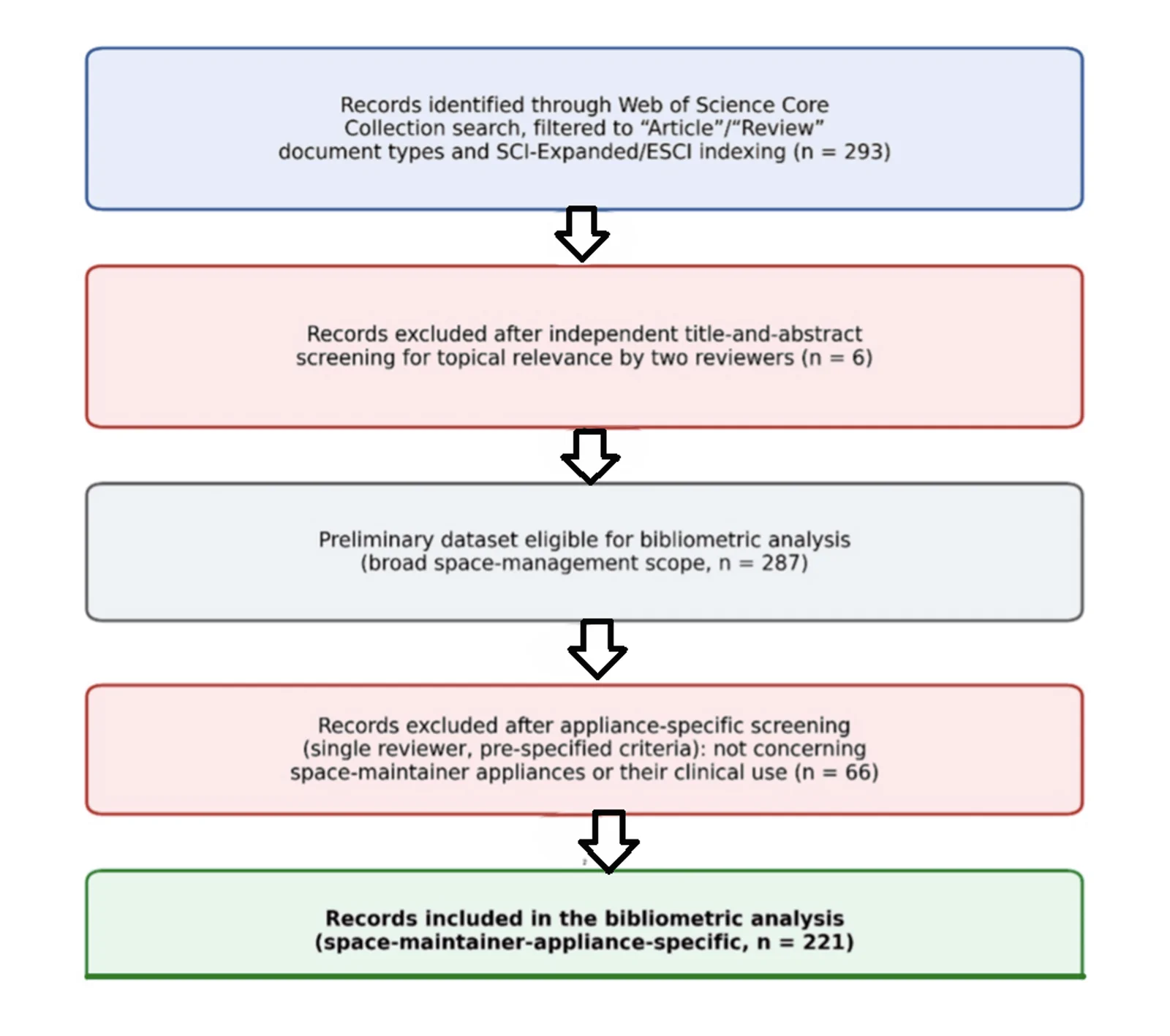
Study selection flow diagram

Bibliometric analysis and software tools

The resulting dataset was analyzed and visualized using bibliometric software tools. Bibliometric analyses were performed using the Bibliometrix package in R (version 4.6.0) through its Biblioshiny interface [15], together with VOSviewer (version 1.6.20). Descriptive bibliometric metrics, including annual scientific production trends, the most productive authors, the most productive countries, and the core journals most frequently addressing the topic, were calculated. Traditional bibliometric laws - Lotka's Law for author productivity [18] and Bradford's Law for journal distribution [19] were used to evaluate the structure of the literature. For Bradford's Law, journals were ranked by decreasing productivity and partitioned into three zones of approximately equal cumulative document count (Core, Zone 2, and Zone 3); the Bradford multiplier k was calculated as the ratio of the number of journals in each successive zone (Zone 2/Core and Zone 3/Zone 2). A keyword co-occurrence analysis was performed using VOSviewer to map the conceptual structure and identify emerging clinical trends. This analysis and the trend-topic analysis presented later were based on author keywords only; Keywords Plus terms were not included. Keywords Plus is generated algorithmically by WoS from words recurring in the titles of an article's cited references and therefore reflects the citation environment of a publication rather than the terminology deliberately assigned by its authors. Its inclusion would have introduced terms that the authors themselves did not select and would have distorted the trend-topic timeline, which is intended to track the evolution of author terminology. A minimum occurrence threshold of three was applied to reduce visual complexity; 38 author keywords met this threshold. Prior to network construction, synonymous terms, singular/plural variations, and spelling variants were standardized using a customized thesaurus file. The thematic descriptions assigned to the identified clusters were interpretive and were not generated automatically by VOSviewer. They were determined by the authors based on the most frequent and conceptually related keywords within each cluster and were intended to facilitate interpretation of the co-occurrence network rather than to define definitive research themes.

Data cleaning and standardization

Prior to the bibliometric analysis, author names, country names, institution names and keywords in the dataset were standardized. In the country analyses, "Turkey" and "Türkiye" were merged as the same country. Author names were standardized by merging abbreviated and fully spelled forms of the same author into a single entry ("Yeluri, R." with "Yeluri, Ramakrishna"; "Nouri, M. R." with "Nouri, M-Reza"; "Qudeimat, MA" with "Qudeimat, M. A."; "Kennedy, D. B." with "Kennedy, David B."), so that author productivity was not underestimated by name-form variation.

The customized thesaurus file was applied exclusively to the author-keyword co-occurrence analysis and the trend-topic analysis; it was not applied to the country, author, journal or citation analyses. Three standardization rules were applied in the following order: (i) singular/plural and synonym unification of the same concept; (ii) abbreviation-expansion unification; and (iii) correction of misspellings and run-together forms present in the source records. Applying these rules, the thesaurus entries were as follows, each given as the standardized term followed by the variants merged into it: space maintainer (space maintainers, space maintenance, space maintanance, spacemaintainers); premature loss (premature tooth loss); children (child); 3D printing (three-dimensional printing); CAD/CAM (cad-cam); and interceptive orthodontics (a lower-casing variant described below).

The final entry requires explanation. When keyword labels were converted to lower case under a Turkish system locale in the analysis software, the capital letter "I" was rendered as the dotless "ı" ("Interceptive orthodontics" appearing as "ınterceptive orthodontics"); such variants were merged with their correctly cased counterparts rather than retained as separate labels.

Compound appliance labels were deliberately not collapsed into their shorter forms, and terms denoting clinically distinct entities were not merged even where semantically adjacent: "band and loop space maintainer" was retained separately from "band and loop," and "fixed space maintainer," "removable space maintainer" and "functional space maintainer" were each retained as separate labels, as was "CAD/CAM" relative to "3D printing," because merging them would have obscured the thematic separation between the clusters.

## Results

A total of 221 publications on space-maintainer appliances in pediatric dentistry, published between 1982 and 2026, met the appliance-specific inclusion criteria; an overview of the main bibliometric parameters of this dataset is presented in Table 1, followed by detailed findings on annual production, country and journal distribution, author productivity, keyword co-occurrence, and citation

**Table 1:** Summary of bibliometric parameters

Parameter	Value
Time span	1982–2026
Total number of publications	221
Document types	Article, Review
Database	Web of Science Core Collection
Bibliometric analysis / visualization tools	Biblioshiny(Bibliometrix/R), VOSviewer
Highest annual output	2021
Most recent trend term	3D printing
Most productive country	India
Rank of Türkiye among most productive countries	2nd

Distribution of research about space maintainers in pediatric dentistry by year

The chronological distribution of the 221 documents meeting the inclusion criteria between 1982 and 2026 is presented in Figure 2.

**Figure 2:**
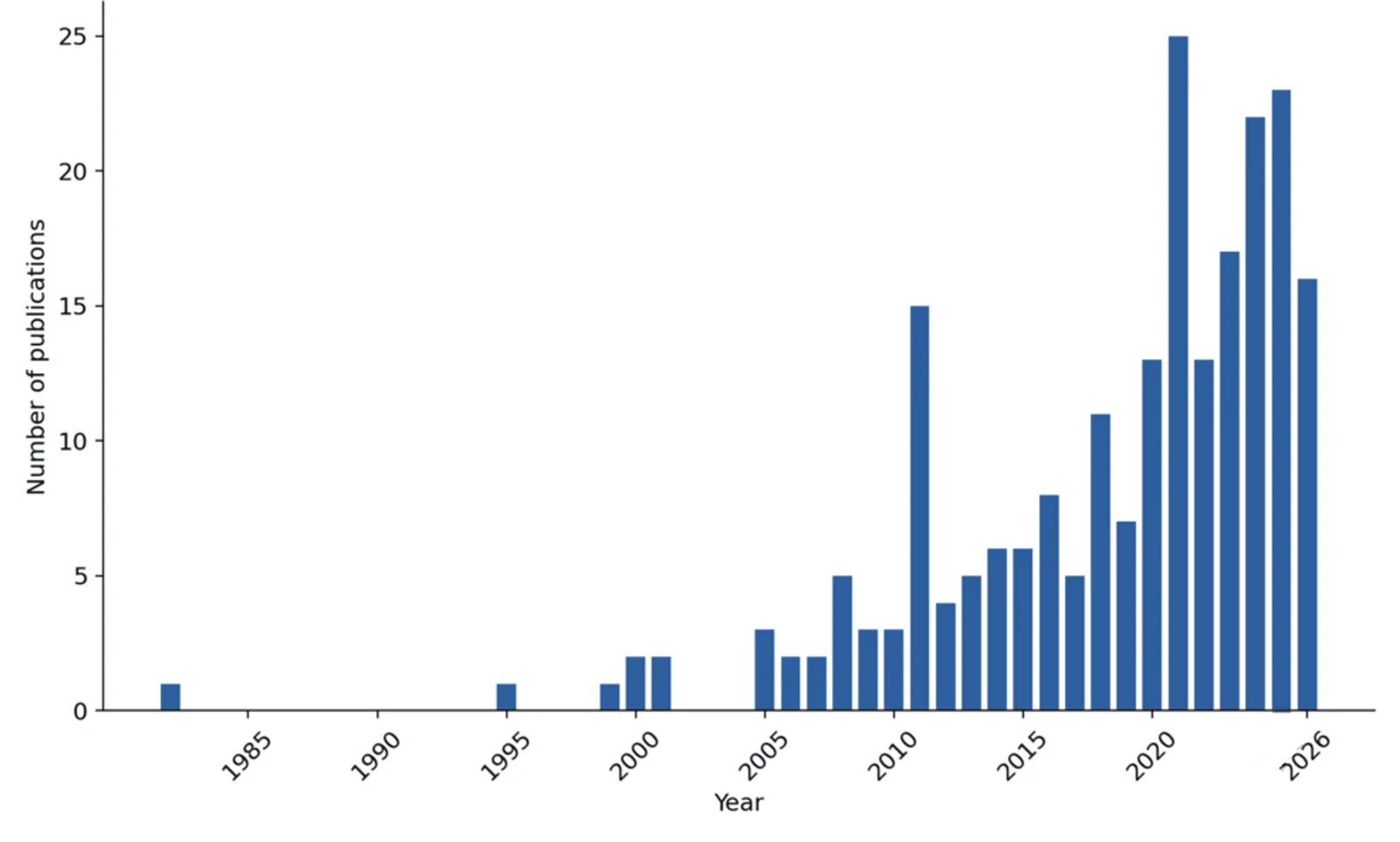
Distribution of research about space maintainers in pediatric dentistry by year (1982–2026) 2026 reflects partial-year data up to 17 June 2026.

The first publication in the literature appeared in 1982 (one article), and no further publications specific to space-maintainer appliances were recorded in the database until 1995. Between 1995 and 2010, publications appeared sporadically, with annual output not exceeding five articles. Starting in 2011, an interim peak of 15 articles was reached. Between 2012 and 2018, the annual number of articles fluctuated between four and 11. From 2019 onward, the literature entered a steep upward trend, reaching its highest historical publication volume with 25 articles in 2021. In the years following the 2021 peak, production remained at a high level, reaching 23 articles in 2025. As of the data collection date, the number of articles indexed in 2026 was 16. Data for 2026 represent partial-year output up to 17 June 2026 and should not be directly compared with complete calendar years.

Distribution of research about space maintainers in pediatric dentistry by country

Full counting analysis of the WoS database was used to determine the scientific production of countries. To avoid duplicate or missing counts that may arise from technical differences in the corresponding author address (RP) matching algorithm of the Biblioshiny software, country production statistics were obtained directly from the WoS "Analyze Results" tool.

The publication volumes of the top 10 contributing countries are presented in Figure 3 and Table 2.

**Figure 3:**
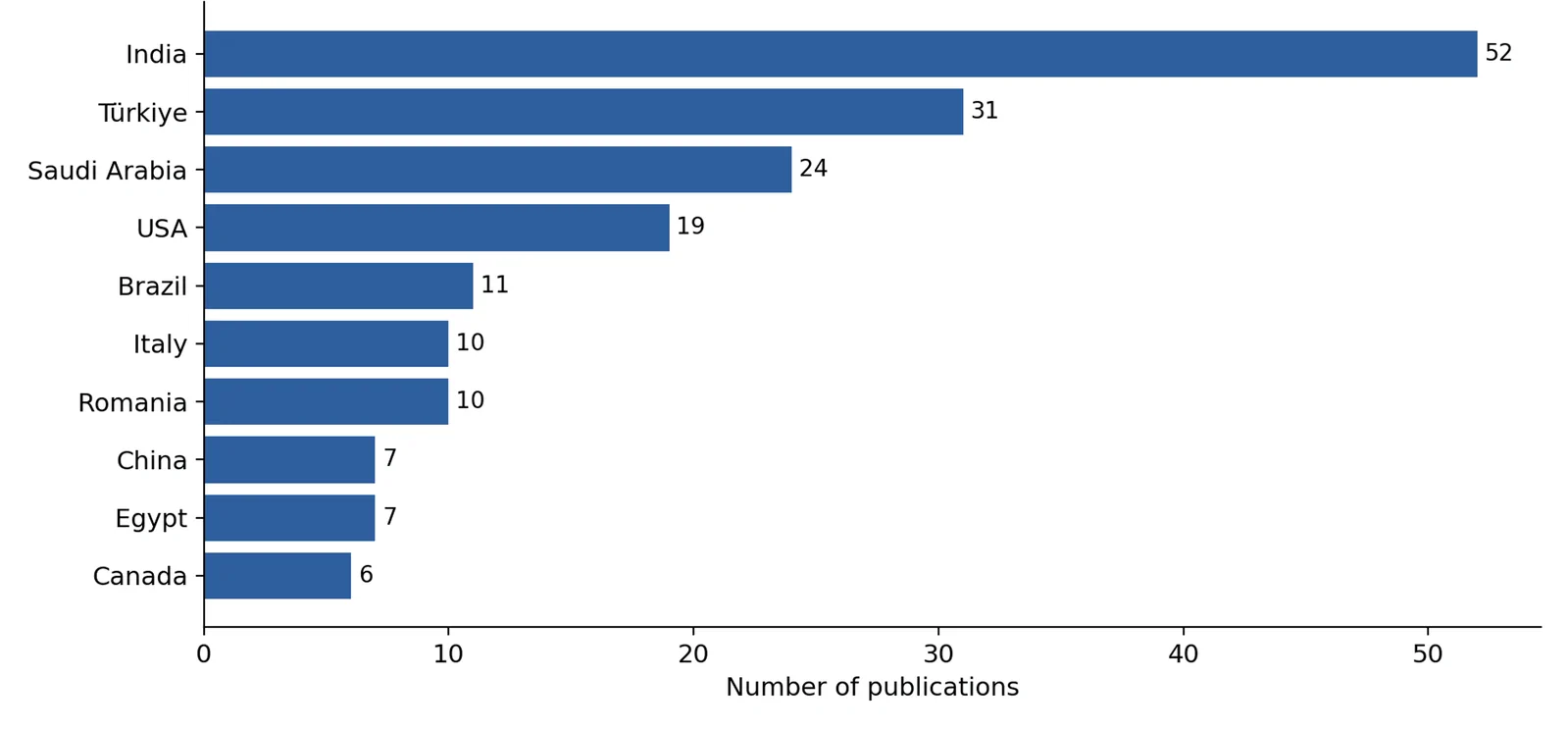
Distribution of research about space maintainers in pediatric dentistry by country

**Table 2:** Distribution of research about space maintainer in pediatric dentistry by country Percentages were calculated using 221 as the denominator. Full counting was applied; therefore, publications with authors from multiple countries were counted once for each contributing country, and country-level percentages are not mutually exclusive and may not sum to 100%.

Rank	Countries/Regions	Record Count	% of 221
1	India	52	23.529
2	Türkiye	31	14.027
3	Saudi Arabia	24	10.860
4	USA	19	8.597
5	Brazil	11	4.977
6	Italy	10	4.525
7	Romania	10	4.525
8	People's Republic of China	7	3.167
9	Egypt	7	3.167
10	Canada	6	2.715

According to the data obtained, India was the most productive country in the field of space maintainers in pediatric dentistry, with 52 articles. After standardization of country names, India was followed by Türkiye (31 articles) and Saudi Arabia (24 articles), while the USA ranked fourth (19 articles).

Publications were recorded in the database under two different name variants, "Turkey" (15 articles) and "Türkiye" (16 articles). When these were merged into a single standardized entry, Türkiye ranked as the second most productive country globally, with a total of 31 articles, immediately behind India. Türkiye was followed by Saudi Arabia, the USA, Brazil, Italy, and Romania.

Keyword co-occurrence network of literature about space maintainers in pediatric dentistry

The co-occurrence analysis of author keywords extracted from the analyzed documents on space maintainers in pediatric dentistry is presented in Figure 4.

**Figure 4:**
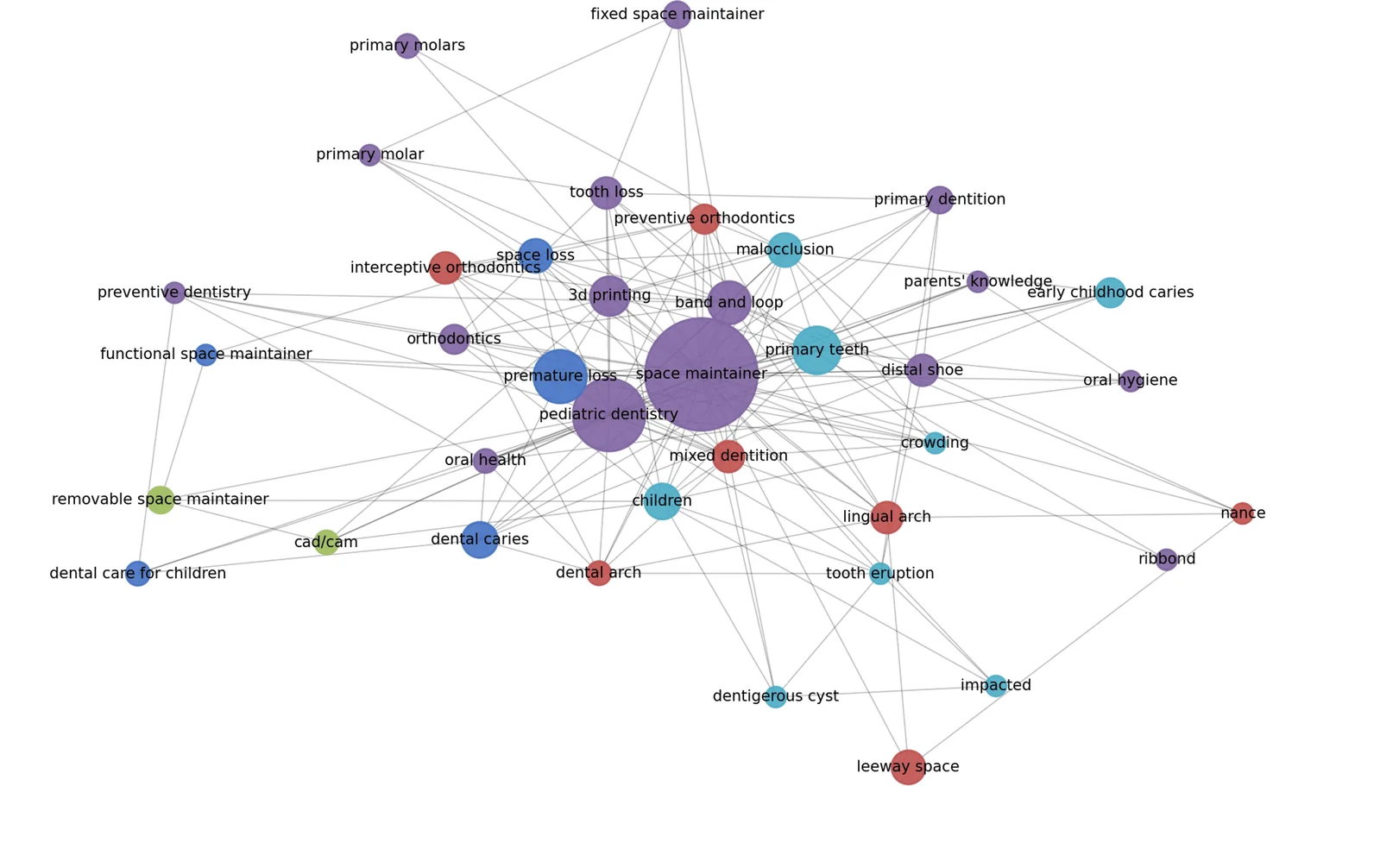
Keyword co-occurrence network of literature about space maintainers in pediatric dentistry

The analysis was computed using a customized thesaurus file. Based on a minimum keyword occurrence threshold of three, the network map consists of five distinct clusters containing a total of 38 interconnected keywords. Node size represents the occurrence frequency of the keywords, while the lines represent the co-occurrence relationships between the terms.

The analysis identified "space maintainer" (89 occurrences), "pediatric dentistry" (37 occurrences), and "premature loss" (20 occurrences) as the keywords with the highest usage frequencies.

The network comprised five software-generated clusters containing 38 interconnected author keywords. Cluster one was the core cluster of literature on space maintainers, centered on space maintainer (89 occurrences), pediatric dentistry (37 occurrences), primary dentition, 3D printing (11 occurrences), distal shoe (seven occurrences), oral health, oral hygiene, orthodontics, preventive dentistry, parents' knowledge, band and loop (13 occurrences), fixed space maintainer (five occurrences), primary molar(s), tooth loss (seveb occurrences), and Ribbond, spanning both conventional fixed appliances and digital fabrication terminology.

Cluster two comprised primary teeth (16 occurrences), children (nine occurrences), malocclusion (eight occurrences), early childhood caries (six occurrences), crowding, tooth eruption, impacted, and dentigerous cyst, reflecting mixed-dentition period dynamics and caries-related outcomes.

Cluster three comprised interceptive orthodontics (seven occurrences), preventive orthodontics (six occurrences), lingual arch (seven occurrences), Nance, dental arch, mixed dentition (seven occurrences), and leeway space (eight occurrences), reflecting active and interceptive appliance use in the mixed dentition.

Cluster four comprised premature loss (20 occurrences), space loss (eight occurrences), dental caries (nine occurrences), functional space maintainer, and dental care for children, reflecting the conventional clinical consequences of early tooth loss.

Cluster five, the smallest, comprised removable space maintainer (five occurrences) and CAD/CAM (four occurrences), reflecting removable and digitally fabricated appliance technologies. A thematic summary of the principal keyword groupings is presented in Table 3.

**Table 3:** Thematic clusters identified by keyword co-occurrence analysis CAD/CAM: computer-aided design/computer-aided manufacturing.

Cluster	Main terms	İnterpretive thematic description
Cluster 1 (Red)	space maintainer, band and loop, fixed space maintainer, primary molar(s), tooth loss, primary dentition, pediatric dentistry, 3D printing, distal shoe, oral health, oral hygiene, orthodontics, preventive dentistry, parents' knowledge, Ribbond	Core space-maintainer terminology, spanning conventional fixed appliances and digital fabrication
Cluster 2 (Green)	primary teeth, children, malocclusion, early childhood caries, crowding, tooth eruption, impacted, dentigerous cyst	Mixed-dentition period dynamics and caries-related outcomes
Cluster 3 (Dark Blue)	interceptive orthodontics, preventive orthodontics, lingual arch, Nance, dental arch, mixed dentition, leeway space	Active and interceptive appliance use in the mixed dentition
Cluster 4 (Yellow)	premature loss, space loss, dental caries, functional space maintainer, dental care for children	Conventional clinical consequences of early tooth loss
Cluster 5 (Purple)	removable space maintainer, CAD/CAM	Removable and digitally fabricated appliances

Trend topic distribution of research about space maintainers in pediatric dentistry

The temporal evolution and frequency distribution of author keywords in the literature are presented in Figure 5.

**Figure 5:**
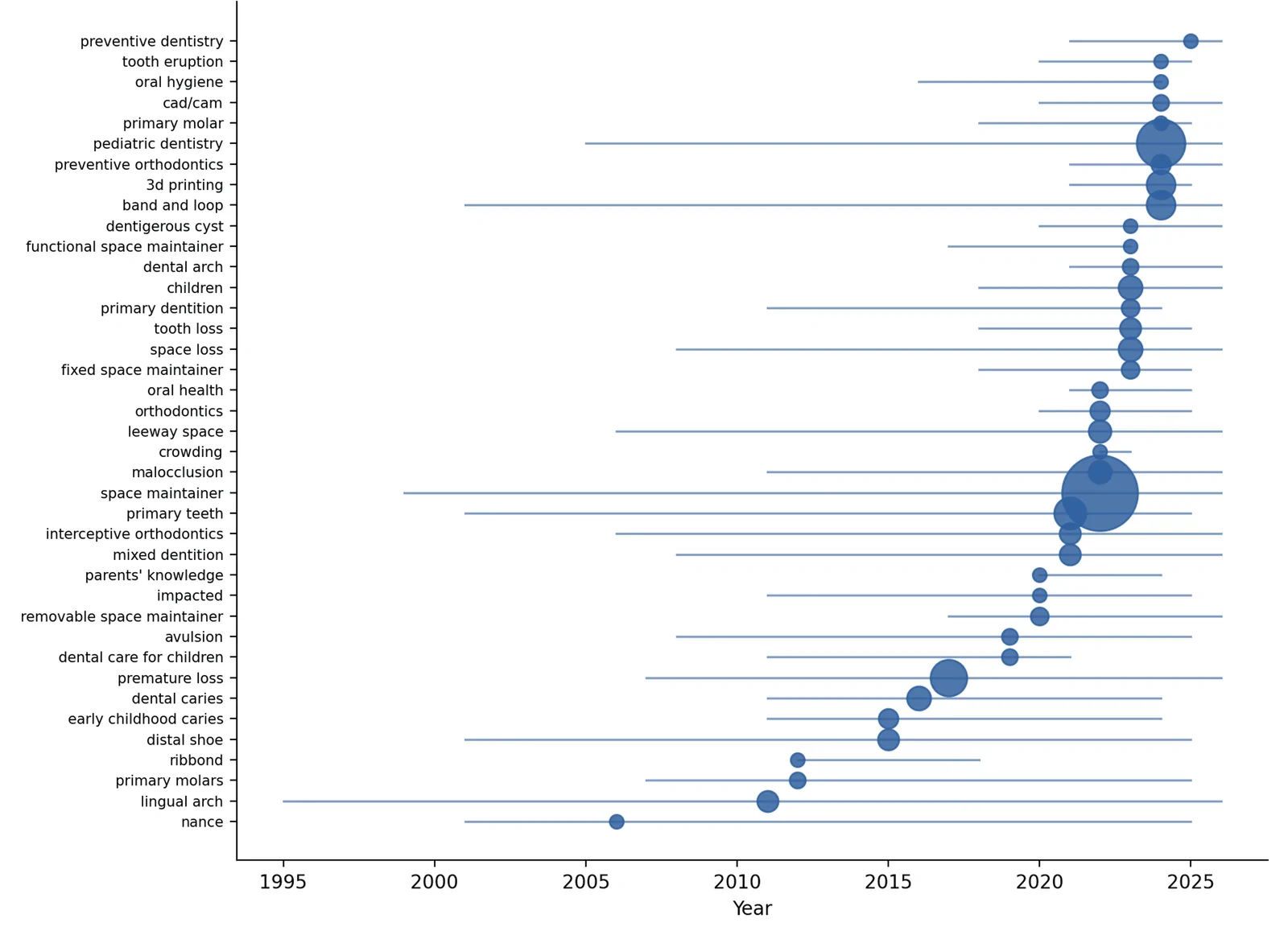
Trend topic distribution of research about space maintainers in pediatric dentistry (1996–2026)

The length of the horizontal lines represents the years during which each keyword was active in the literature, while the size of the blue circles at the midpoint of each line represents the total frequency of use of that term.

Examination of the chronological flow showed that in the early period (up to 2015), the terms "Nance," "lingual arch," "primary molars," "Ribbond," "distal shoe," and "early childhood caries" were prominent. In the intermediate period (2016-2020), the keywords "dental caries," "premature loss," "avulsion," "removable space maintainer," and "impacted" appeared in the literature. In the recent period, covering 2021 onward, the core terms with the highest frequency of use (largest circles) were "space maintainer," "children," "pediatric dentistry," and "band and loop. “The recent literature showed increasing prominence of the keywords ‘3D printing,’ ‘CAD/CAM,’ and ‘preventive orthodontics,’ particularly during the 2021-2025 period, indicating emerging research themes within the space-maintainer literature.”

Authors contributing most to research about space maintainers in pediatric dentistry

The top 10 individual researchers who contributed the most publications to the 221 documents in the dataset, along with their net article counts, are presented in Figure 6.

**Figure 6:**
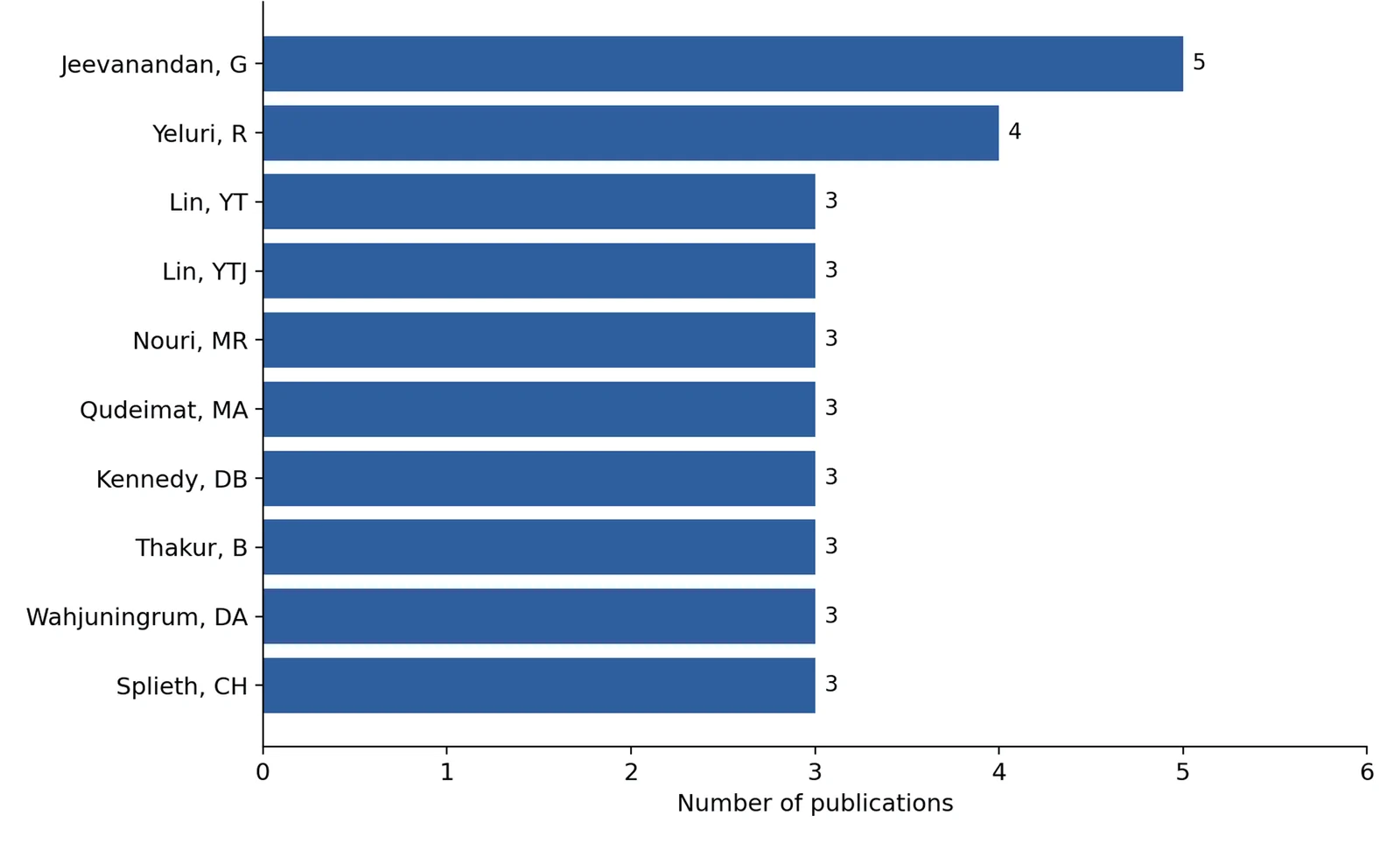
Top 10 authors contributing most to research about space maintainers in pediatric dentistry

According to the findings of the descriptive analysis, the most productive author in the field of space maintainers in pediatric dentistry was Jeevanandan, Ganesh, with five articles. This researcher was followed by Yeluri, Ramakrishna, with four articles. The remaining eight authors in the ranking (Lin, Yai-Tin; Lin, Yng-Tzer J.; Nouri, M-Reza; Qudeimat, M. A.; Kennedy, David B.; Thakur, Bhagyashree; Wahjuningrum, Dian Agustin; and Splieth, Christian H.) each contributed three articles.

The mathematical frequency distribution of author scientific productivity in the dataset and its conformity to Lotka's Law are presented in Figure 7.

**Figure 7:**
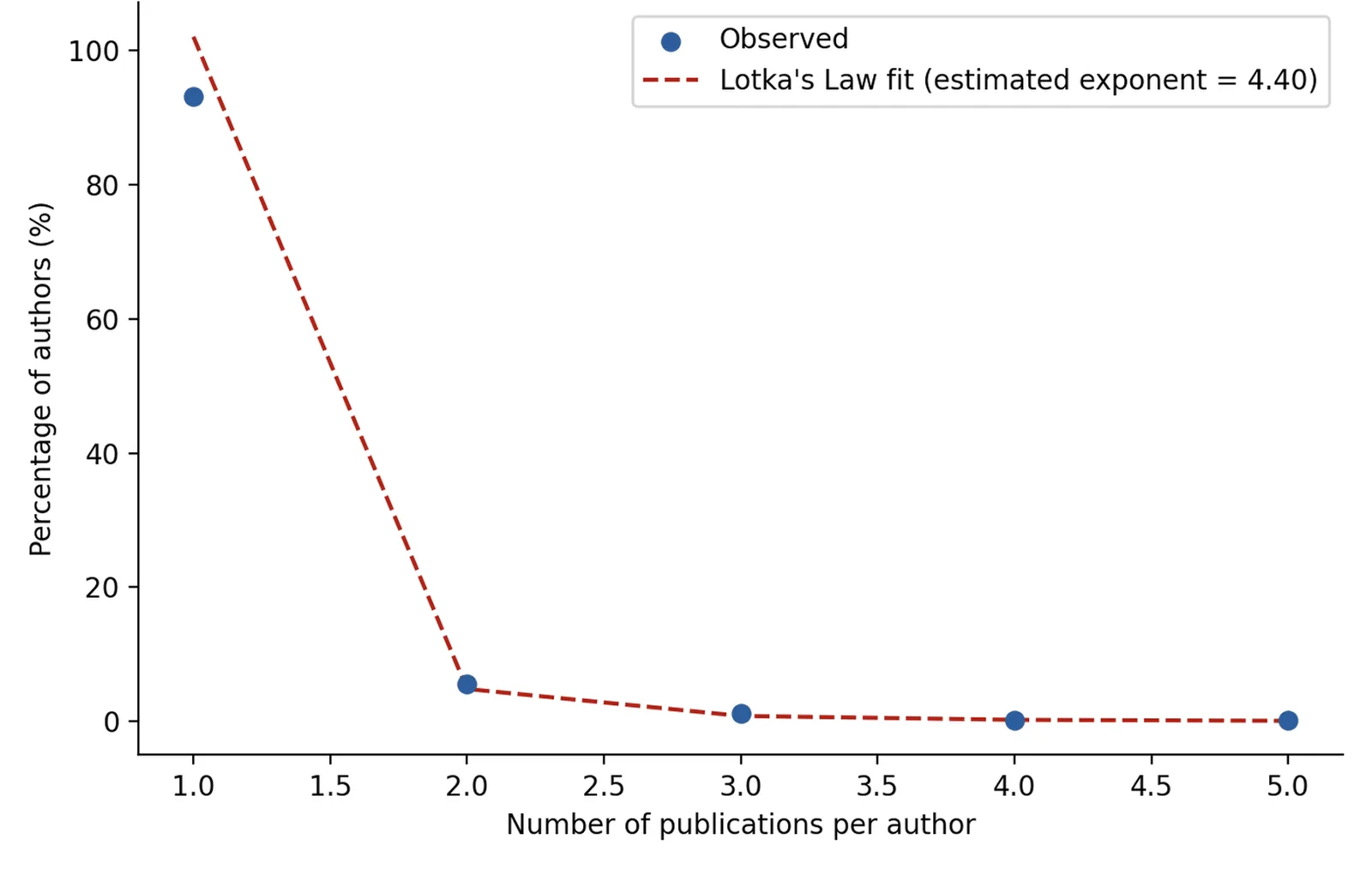
Distribution of author productivity for research about space maintainers in pediatric dentistry according to Lotka's Law

The theoretical curve was fitted by ordinary least-squares regression on the log-transformed observed distribution of author productivity, yielding an estimated Lotka exponent of 4.40. According to the Kolmogorov-Smirnov (KS) goodness-of-fit test comparing the observed and fitted distributions, the test statistic was KS = 0.200 (asymptotic p=1.000; p>0.05), and the coefficient of determination was R²=0.9995. These statistical values suggest good agreement between the literature about space maintainers in pediatric dentistry and the theoretical author productivity model of Lotka's Law, although the small number of distinct productivity levels in this dataset (authors publishing one to five articles) limits the discriminatory power of the KS test and should be interpreted with appropriate caution.

Examination of the distribution curve shows that the vast majority of authors in this field (93.1%) contributed to the literature with only a single or one article. The proportion of authors who published two articles decreased sharply to 5.5%. The core group of researchers with three or more articles constitutes the asymptotic tail at the far right of the curve, representing a very small percentage of the total author population.

Journals publishing the most research on space maintainers in pediatric dentistry

The top 10 most relevant sources in which the analyzed 221 documents were published, along with the number of articles published in each, are presented in Figure 8 and Table 4.

**Figure 8:**
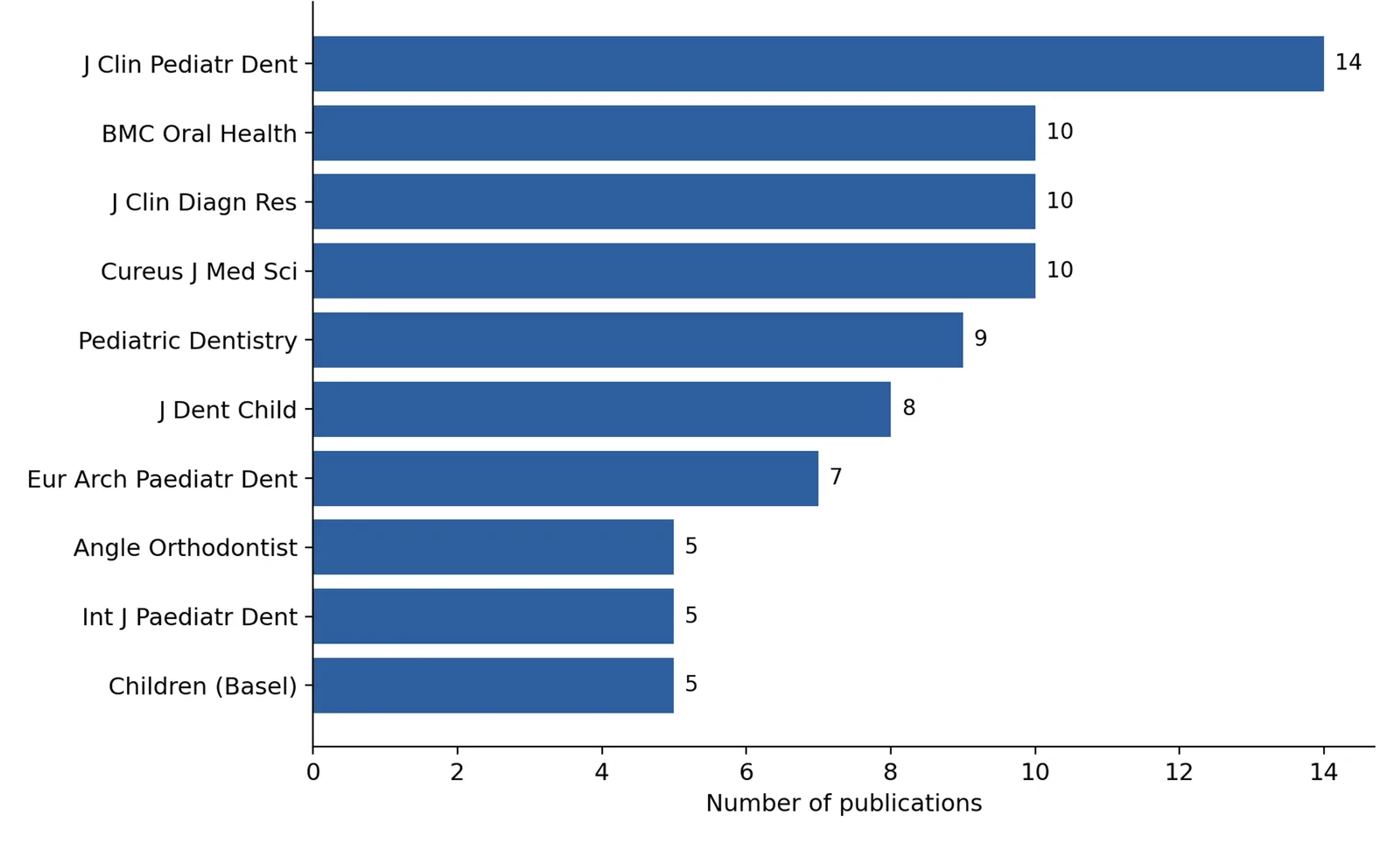
Top 10 journals publishing the most research on space maintainers in pediatric dentistry

**Table 4:** Most relevant sources

Source	Articles
Journal of Clinical Pediatric Dentistry	14
BMC Oral Health	10
Journal of Clinical and Diagnostic Research	10
Cureus Journal of Medical Science	10
Pediatric Dentistry	9
Journal of Dentistry for Children	8
European Archives of Paediatric Dentistry	7
Angle Orthodontist	5
International Journal of Paediatric Dentistry	5
Children (Basel)	5

According to the findings, the leading journal with the highest number of publications on space maintainers in pediatric dentistry was the Journal of Clinical Pediatric Dentistry, with 14 articles. This source was followed, respectively, by BMC Oral Health, the Journal of Clinical and Diagnostic Research, and Cureus Journal of Medical Science, each with 10 articles. Among the other journals in the ranking, Pediatric Dentistry contributed nine articles, and the Journal of Dentistry for Children and the European Archives of Paediatric Dentistry contributed eight and seven articles, respectively. At the end of the list, the Angle Orthodontist, the International Journal of Paediatric Dentistry, and Children (Basel) each contributed five articles to the literature on space maintainers in pediatric dentistry.

The mathematical distribution of the analyzed 221 documents according to their journals (sources) and their conformity to Bradford's Law are presented in Figure 9.

**Figure 9:**
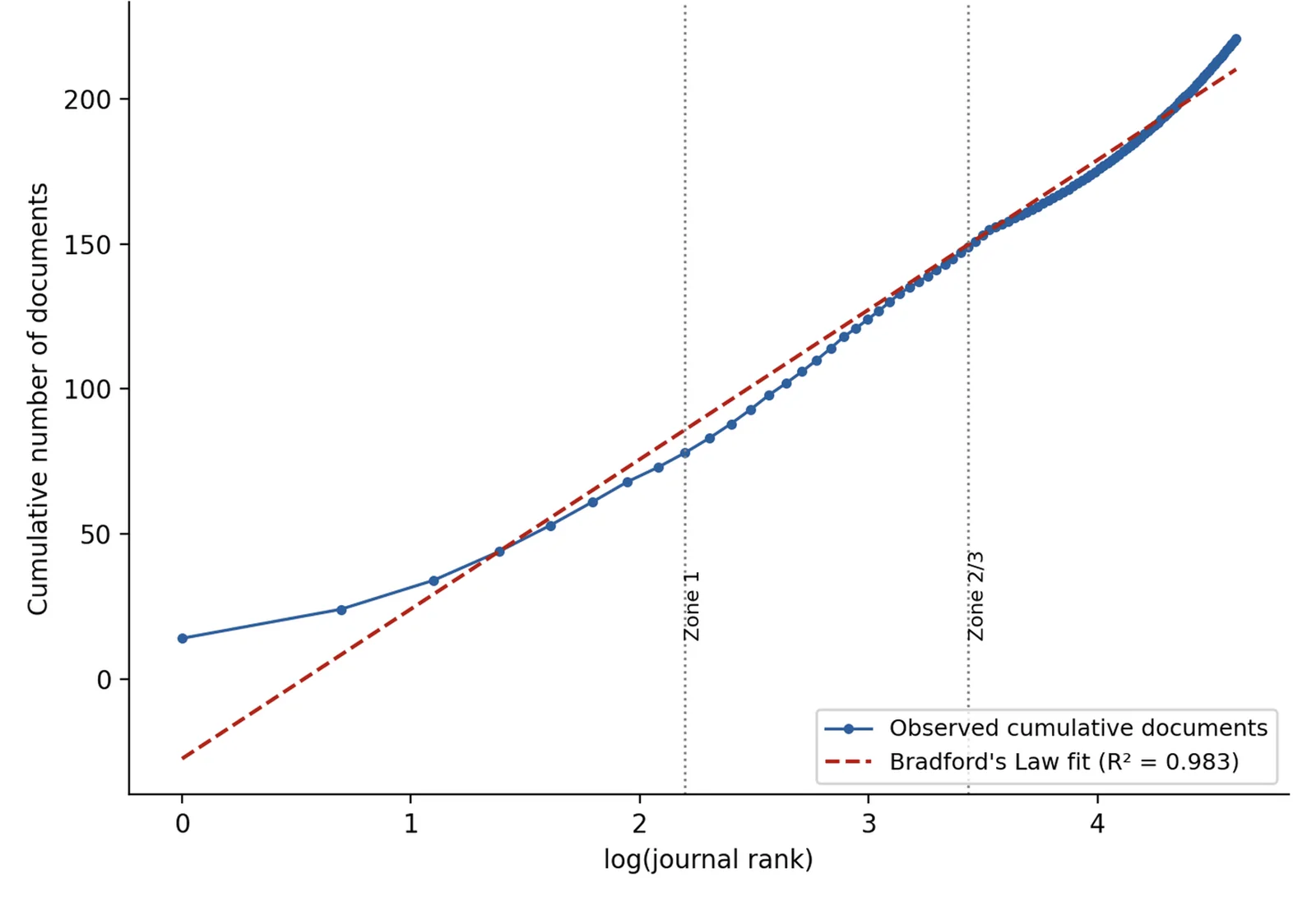
Journal distribution model of publications on space maintainers in pediatric dentistry according to Bradford's Law

According to the KS goodness-of-fit test results, the calculated p-value (KS p-value = 0.368; p>0.05) and coefficient of determination (R²=0.983) were obtained. The Bradford multiplier was calculated as k ≈ 2.4-3.1 across successive zones. These values suggest that the distribution of the literature about space maintainers was broadly consistent with the theoretical Bradford's Law model. According to the Bradford model, sources were divided into three main zones that equally share the total number of publications. The first zone (Core Zone) consists of nine core sources (journals) that alone account for one-third of the articles on space maintainers in pediatric dentistry. The second zone (Zone 2) includes 22 sources representing more peripheral publication outlets, while the third zone (Zone 3) contains 69 sources that publish infrequently or are more distant from the field.

Top 10 most-cited publications in the final appliance-specific dataset, classified according to their primary research focus 

The highly cited publications comprised both studies directly focused on space-maintainer appliances and studies addressing broader space-management outcomes. The latter were retained because they met the predefined appliance-specific and space-management eligibility criteria, although their primary outcomes included arch-length changes, crowding, or other orthodontic consequences of space preservation rather than appliance performance itself.

The 10 most cited publications in the final dataset showed two broad patterns (Table 5).

**Table 5:** Top 10 most-cited publications in the final appliance-specific dataset, classified according to their primary research focus Publications classified as “Broader space-management/orthodontic outcome” were retained because they fulfilled the predefined appliance-specific and space-management-context eligibility criteria, although their primary outcomes concerned broader orthodontic or space-related changes.

Rank	Author and Year	Article Title	Journal	Primary Research Focus	Classification	Total Citations
1	Dugoni et al., 1995 [20]	Early mixed dentition treatment: postretention evaluation of stability and relapse	Angle Orthodontist	Long-term orthodontic outcomes following early mixed-dentition treatment	Broder space-management/orthodontic outcome	65
2	Ramakrishnan et al., 2019 [21]	Survival rate of different fixed posterior space maintainers used in paediatric dentistry — a systematic review	The Saudi Dental Journal	Survival of fixed space maintainer appliances	Direct space maintainer research	62
3	Brennan et al., 2000 [22]	The use of the lingual arch in the mixed dentition to resolve incisor crowding	American Journal of Orthodontics and Dentofacial Orthopedics	Crowding resolution associated with lingual arch therapy	Broader orthodontic outcome associated with a space–maintainer appliance	54
4	Tunison et al., 2008 [23]	Dental arch space changes following premature loss of primary first molars: a systematic review	Pediatric Dentistry	Changes in dental arch space following premature primary molar loss	Broader space-management outcome	48
5	Terlaje et al., 2001 [24]	Treatment planning for space maintenance in the primary and mixed dentition	Journal of Dentistry for Children	Treatment planning for space maintenance	Direct space-maintainer research	30
6	Northway, 2000 [25]	The not-so-harmless maxillary primary first molar extraction	Journal of the American Dental Association	Orthodontic and space-related consequences of primary molar extraction	Broader space-management outcome	30
7	Lin et al., 2007 [26]	Immediate and six-month space changes after premature loss of a primary maxillary first molar	Journal of the American Dental Association	Space changes following premature primary molar loss	Broader space-management outcome	29
8	Sonis et al., 2011 [27]	E-space preservation: is there a relationship to mandibular second molar impaction?	Angle Orthodontist	E-space preservation and subsequent eruption/impaction outcomes	Broader space-management/orthodontic outcome	29
9	Watt et al., 2018 [28]	Space maintainers in the primary and mixed dentition — a clinical guide	British Dental Journal	Clinical use and management of space-maintainer appliances	Direct space-maintaner research	28
10	Khanna et al., 2021 [29]	3D printed band and loop space maintainer: a digital game changer in preventive orthodontics	Journal of Clinical Pediatric Dentistry	Digital fabrication of a band-and-loop space maintainer	Direct space-maintainer research	28

Four publications primarily focused on space-maintainer appliances, including appliance survival, treatment planning, clinical guidance, and digital fabrication. The remaining six highly cited publications addressed broader space-management or orthodontic outcomes, such as crowding, arch-length changes, premature molar loss, E-space preservation, and post-treatment stability. These studies were retained because they fulfilled the predefined appliance-specific and space-management-context eligibility criteria. Accordingly, their inclusion reflects the broader scope of space-management research captured by the search strategy rather than direct evidence of space-maintainer performance.

## Discussion

This bibliometric analysis demonstrates a substantial expansion of research on pediatric space maintainers in recent years, accompanied by increasing attention to digital fabrication technologies. The findings suggest that the field is evolving from a predominantly conventional appliance-focused research area toward a broader research landscape that increasingly incorporates digital workflows and fabrication technologies.

The prominent contribution of India and Türkiye indicates that research activity on pediatric space maintainers is particularly concentrated in these countries. The identification of core journals and the observed distribution of author productivity further demonstrated recognizable structural patterns within the literature. However, these bibliometric patterns should be interpreted as characteristics of publication activity rather than direct indicators of research quality or clinical advancement.

Space maintainers are orthodontic appliances used to prevent space loss resulting from the early loss of primary teeth [30]. Conventionally fabricated space maintainers have reported disadvantages such as adverse effects on gingival health, limited survival time, wire bending, and cement loss, which has led researchers to seek alternative fabrication methods [31]. With the growing popularity of intraoral scanners and CAD/CAM technology in dentistry, individual clinical studies have reported that the number of appointments may decrease and that space-maintainer therapy may become more feasible for children with metal allergies through the use of biocompatible materials compatible with 3D printing technology [32]. However, these are findings from individual clinical studies rather than conclusions of the present bibliometric analysis.

This study maps the increasing visibility of 3D printing technology within the literature about space maintainers in pediatric dentistry. The temporal and frequency patterns observed in the keyword analysis indicate growing research attention to digital fabrication, but do not by themselves establish clinical adoption or superiority. In this study, which analyzed articles and reviews published between 1982 and 2026, researchers' interest in digitally fabricated space maintainers appears to have increased in parallel with the broader adoption of 3D printing technologies in pediatric dentistry. The increasing research activity observed in recent years coincides temporally with the broader adoption of digital workflows in dentistry. The availability of intraoral scanning, CAD/CAM technologies, and 3D printing may have contributed to this development by facilitating digital appliance design and fabrication. However, this temporal association should be interpreted as a possible explanation rather than evidence of a causal relationship, as the present bibliometric analysis cannot determine the factors driving publication growth.This observation is consistent with systematic evidence confirming the high dimensional accuracy and reproducibility of customized 3D-printed orthodontic appliances relative to conventional designs [33].

In our study, the co-occurrence of “3D printing” within the same cluster as “space maintainer,” “band and loop,” and other core conventional fixed-appliance terms (Cluster one) suggests that digital fabrication is currently being investigated alongside, rather than as a separate research stream from, the established literature on conventional fixed appliances. The smaller and more distinct grouping of “removable space maintainer” and “CAD/CAM” (Cluster five) indicates a more specific thematic association between removable appliance design and digital workflows. These findings reflect patterns of keyword co-occurrence within the literature and should therefore be interpreted as indicators of research trends rather than evidence of clinical effectiveness or superiority.

Independent clinical and laboratory studies have nevertheless investigated specific performance characteristics of digitally fabricated space maintainers. Metkar et al. reported that space maintainers produced using 3D printing technology exhibited higher fracture strength than conventionally fabricated space maintainers [34]. Abdelshahed et al. reported that polyetheretherketone (PEEK) space maintainers fabricated using 3D printing technology demonstrated better clinical performance than conventionally fabricated space maintainers at the nine-month follow-up [35]. Similarly, Guo et al. found that removable space maintainers fabricated using 3D printing technology showed better fit to dental models than conventionally fabricated removable space maintainers [36]. These clinical and laboratory findings provide complementary evidence regarding specific properties of digitally fabricated appliances; however, they should be considered separately from the bibliometric findings of the present study, which describe the evolution and thematic structure of the research literature rather than establishing clinical superiority.

Khanna et al. reported that space maintainers fabricated with the 3D printing method showed good fit, were a less invasive method, and reduced chair time and the number of laboratory steps [29]. Similarly, Khan et al., in a study on the space maintainer literature in pediatric patients, reported - based on sources obtained from various databases - that space maintainers fabricated with 3D printing technology were more successful in terms of patient and clinician comfort than conventionally fabricated space maintainers [37].

These reported findings may have contributed to increased research interest in digital fabrication; however, the present bibliometric analysis cannot determine whether specific clinical advantages were responsible for the observed increase in publication activity. As shown in Figure 5, an analysis of the trend topic distribution of research about space maintainers in pediatric dentistry indicated that keyword usage for "3D printing" reached its highest prominence in the 2024-2025 period; this reflects the term's frequency and recency within the author-keyword network rather than the annual count of publications shown in Figure 2.

Clinical implications for pediatric dentistry

The findings of this study indicate that digital technologies are becoming an increasingly important research area for space maintainer applications in pediatric dentistry. Clinical success in pediatric patients depends not only on the mechanical durability of the appliance but also on factors such as patient cooperation, appointment duration, comfort, oral hygiene, material biocompatibility, and traceability [11]. Digital impression systems and 3D printing technologies can facilitate treatment, particularly in young children, those with dental anxiety, or those with limited cooperation, by reducing conventional impression-taking procedures [9,37]. In addition, digital archiving enables faster and more standardized remanufacturing in cases of appliance fracture or loss [11,21]. However, the current bibliometric findings do not directly prove the clinical superiority of these technologies; therefore, they need to be supported by long-term clinical studies.

Evidence gaps and future research directions

Although interest in 3D-printed and CAD/CAM-assisted space maintainers has increased in the literature, the level of evidence regarding the long-term clinical success of these technologies remains limited [12,25]. Future studies should evaluate clinical outcomes such as appliance survival, cement loss, fracture strength, plaque retention, periodontal effects, patient satisfaction, child cooperation, material biocompatibility, and cost-effectiveness [31,34]. In particular, randomized controlled clinical trials and long-term follow-up studies are important for demonstrating the real clinical advantages of digitally fabricated space maintainers over conventional methods [31,35].

Strengths

This study has several notable strengths. To the best of our knowledge, few bibliometric studies have specifically mapped the research landscape of space maintainers in pediatric dentistry. The present study provides a comprehensive bibliometric assessment covering the indexed literature from 1982 to 2026 and combines descriptive bibliometric indicators, keyword co-occurrence analysis, trend-topic analysis, and evaluation according to Lotka’s and Bradford’s laws. The dataset was restricted to peer-reviewed articles and reviews indexed in SCI-Expanded/ESCI, screened independently by two reviewers for initial topical relevance and subsequently refined through an explicit, appliance-specific screening stage, supporting the specificity of the included records to space-maintainer appliances. Finally, by explicitly tracking the emergence and thematic positioning of "3D printing" and "CAD/CAM" within the keyword network, this study provides an early, data-driven characterization of the emerging digital fabrication research direction in pediatric space-maintainer research, which may serve as a reference point for future updates of this analysis.

Limitations

This study has several limitations. First, the analysis was based exclusively on the WoS Core Collection, which may have introduced database-selection bias. Although WoS provides standardized bibliographic and citation data suitable for reproducible bibliometric analyses, it does not comprehensively index all publications worldwide. Consequently, relevant studies indexed exclusively in Scopus, PubMed/MEDLINE, Embase, or regional bibliographic databases may have been omitted. This limitation may particularly affect the representation of countries, institutions, journals, and emerging research topics with lower or more variable coverage in WoS. Therefore, the findings represent the global research landscape captured by the WoS Core Collection rather than an exhaustive census of the entire worldwide literature. Future studies combining multiple bibliographic databases may provide a broader and more comprehensive representation of the field. Second, bibliometric findings are inherently influenced by database indexing policies, author keyword selection, citation practices, and publication language, with English-language publications being predominant in the WoS database. Third, recently published articles may have accumulated fewer citations because of their shorter exposure time, resulting in citation-age bias; this limitation is particularly relevant to the partial-year 2026 data.

Fourth, because the original search strategy was intentionally broad, an additional appliance-specific screening stage was required to exclude studies on dental trauma, crossbite correction, and maxillary protraction that shared search terminology (e.g., “lingual arch,” “Nance appliance,” or “transpalatal arch”) without concerning space-maintainer therapy itself. Although the initial topical screening was independently performed by two reviewers, the second-stage appliance-specific screening of the 287 preliminary records was conducted by a single reviewer using predefined title/abstract eligibility criteria. Although this approach enabled consistent application of the appliance-specific criteria, the absence of independent duplicate screening at this stage may have introduced reviewer-selection bias, particularly for borderline records involving terms such as “lingual arch,” “Nance appliance,” or “transpalatal arch.” This limitation should be considered when interpreting the composition of the final 221-record dataset. Fifth, the keyword co-occurrence and trend-topic analyses were restricted to author keywords; records without indexed author keywords therefore did not contribute to the network map, and terms appearing only in titles, abstracts or Keywords Plus are not reflected in the conceptual structure reported here.

Finally, although this study identified the emergence of 3D printing and CAD/CAM technologies as important trends in the literature about space maintainers, bibliometric analysis does not directly assess the clinical effectiveness, survival rate, patient satisfaction, biocompatibility, or cost-effectiveness of digitally fabricated space maintainers. Therefore, the increasing bibliometric prominence of these technologies should not be interpreted as evidence of clinical superiority. Further clinical and long-term outcome studies are required to determine their actual clinical benefits and limitations.

## Conclusions

This bibliometric analysis demonstrates a clear increase in research activity on space maintainers in pediatric dentistry, particularly after 2019. India was the most productive country, while Türkiye ranked second, and the literature showed established patterns of author productivity and journal distribution. Keyword analyses indicated that conventional space-maintainer concepts remain central to the field, while 3D printing and CAD/CAM have emerged as increasingly visible topics in recent years. These findings indicate growing research interest in digitally fabricated space maintainers, but do not provide evidence of their clinical effectiveness or superiority over conventional approaches. Further well-designed clinical studies, randomized controlled trials, and long-term follow-up investigations are required to evaluate the clinical performance, durability, patient-related outcomes, and cost-effectiveness of digitally fabricated space maintainers.
